# Utilizing Deep Learning for X-ray Imaging: Detecting and Classifying Degenerative Spinal Conditions

**DOI:** 10.7759/cureus.41582

**Published:** 2023-07-08

**Authors:** Muhammad S Ghauri, Akshay J Reddy, Nathaniel Tak, Ethan A Tabaie, Ajay Ramnot, Parsa Riazi Esfahani, Neel Nawathey, Javed Siddiqi

**Affiliations:** 1 Neurosurgery, California University of Science and Medicine, Colton, USA; 2 Medicine, California University of Science and Medicine, Colton, USA; 3 Medicine, Midwestern University Arizona College of Osteopathic Medicine, Glendale, USA; 4 Medicine, California Northstate University College of Medicine, Elk Grove, USA; 5 Neurosurgery, Desert Regional Medical Center, Palm Springs, USA; 6 Health Sciences, California Northstate University College of Medicine, Elk Grove, USA; 7 Neurosurgery, Riverside University Health System Medical Center, Moreno Valley, USA; 8 Neurosurgery, Arrowhead Regional Medical Center, Colton, USA

**Keywords:** ai and robotics in healthcare, spine injury, artificial intelligence (ai), x ray, degenerative spine disease, deep learning

## Abstract

Background

Degenerative spinal conditions (DSCs) involve a diverse set of pathologies that significantly impact health and quality of life, affecting many individuals at least once during their lifetime. Treatment approaches are varied and complex, reflecting the intricacy of spinal anatomy and kinetics. Diagnosis and management pose challenges, with the accurate detection of lesions further complicated by age-related degeneration and surgical implants. Technological advancements, particularly in artificial intelligence (AI) and deep learning, have demonstrated the potential to enhance detection of spinal lesions. Despite challenges in dataset creation and integration into clinical settings, further research holds promise for improved patient outcomes.

Methods

This study aimed to develop a DSC detection and classification model using a Kaggle dataset of 967 spinal X-ray images at the Department of Neurosurgery of Arrowhead Regional Medical Center, Colton, California, USA. Our entire workflow, including data preprocessing, training, validation, and testing, was performed by utilizing an online-cloud based AI platform. The model's performance was evaluated based on its ability to accurately classify certain DSCs (osteophytes, spinal implants, and foraminal stenosis) and distinguish these from normal X-rays. Evaluation metrics, including accuracy, precision, recall, and confusion matrix, were calculated.

Results

The model achieved an average precision of 0.88, with precision and recall values of 87% and 83.3%, respectively, indicating its high accuracy in classifying DSCs and distinguishing these from normal cases. Sensitivity and specificity values were calculated as 94.12% and 96.68%, respectively. The overall accuracy of the model was calculated to be 89%.

Conclusion

These findings indicate the utility of deep learning algorithms in enhancing early DSC detection and screening. Our platform is a cost-effective tool that demonstrates robust performance given a heterogeneous dataset. However, additional validation studies are required to evaluate the model's generalizability across different populations and optimize its seamless integration into various types of clinical practice.

## Introduction

Degenerative spinal conditions (DSCs) encompass a broad spectrum of conditions that affect the structural elements of the spine that can significantly impact an individual's health and quality of life [1-6]. Globally, it has been estimated that nearly 30% of adults may suffer from a spinal disorder with even more individuals at risk of DSCs sometime in their lifetime. [1]. From degenerative conditions, such as disc disease, foraminal stenosis, and osteophytes, to traumatic events leading to fractures and dislocations, the breadth of DSCs is expansive and their prevalence significant [5].

Treatment approaches to these conditions are as varied as their etiologies. Conservative management strategies, surgical interventions, and novel therapeutic approaches are all part of clinicians’ armamentarium when dealing with these pathologies [2]. Nevertheless, the complexity of spinal anatomy and the subtle nuances of these conditions can pose challenges in diagnosis and management [4].

The detection and characterization of these DSCs can be challenging due to the complexity of the spine's anatomy, presence of age-related deterioration, and subtlety of some DSCs in imaging studies [5,7]. This is particularly true in the presence of surgical implants, which can cause artifacts in images, making it difficult to identify and assess surrounding tissues [3].

Technological advancements in imaging modalities have certainly provided clinicians with improved visualization capabilities. Nonetheless, distinguishing pathological DSCs from normal age-related changes or identifying small or complex DSCs can be a demanding task. Recent studies have highlighted the potential of artificial intelligence (AI) in enhancing image detection for DSCs [8]. Deep learning algorithms, a subset of AI, have demonstrated promising results in identifying and classifying spinal pathologies [9-10]. These computational models, trained on vast datasets of spinal images, show potential to augment diagnostic capabilities, optimize patient management, and ultimately improve patient outcomes [9]. However, further research is needed to fully integrate and validate these technologies within the clinical setting.

Despite these advancements, challenges remain. Deep learning algorithms require large, accurately labeled datasets for training, which can be labor-intensive and costly to produce. Furthermore, the application of these systems in real-world clinical settings poses its own set of challenges, including integrating them into existing workflows and ensuring that they operate reliably and safely [11]. Privacy concerns challenging the ethical implications of AI technology have also been raised [12]. Despite these hurdles, the potential of deep learning in the field of DSC detection is promising, and further research and development is likely to yield significant benefits for all stakeholders in spinal disease management.

## Materials and methods

This study was conducted at the Department of Neurosurgery of Arrowhead Regional Medical Center, Colton, California, USA. It aimed to develop a proof-of-concept DSC detection and classification model using spinal X-ray images obtained from a Kaggle dataset [13]. A dataset comprising over 967 images, including 311 spinal stenosis, 121 normal, 136 spinal implant, and 399 osteophyte cases, was utilized for model development. All images used throughout our workflow were in PNG digital format with 300 x 300 pixels in size. These images were heterogeneous in nature, captured in a variety of imaging planes (i.e., oblique and transverse) and locations (i.e., cervical, thoracic, and lumbar).

Our initial data preprocessing included separating our DSC images and normal images into respective folders. Each image was visually inspected and confirmed to ensure accurate quality and subsequent labeling.

Next, we implemented data processing and augmentation techniques as previously described in the literature [14]. Our model automatically optimized varying combinations of noise injection, pixelation, image translation, cropping, resizing, and zooming to better unify the heterogeneous nature of images. This helps to expand our training dataset, thereby yielding the highest classification accuracy possible.

**Figure 1:**
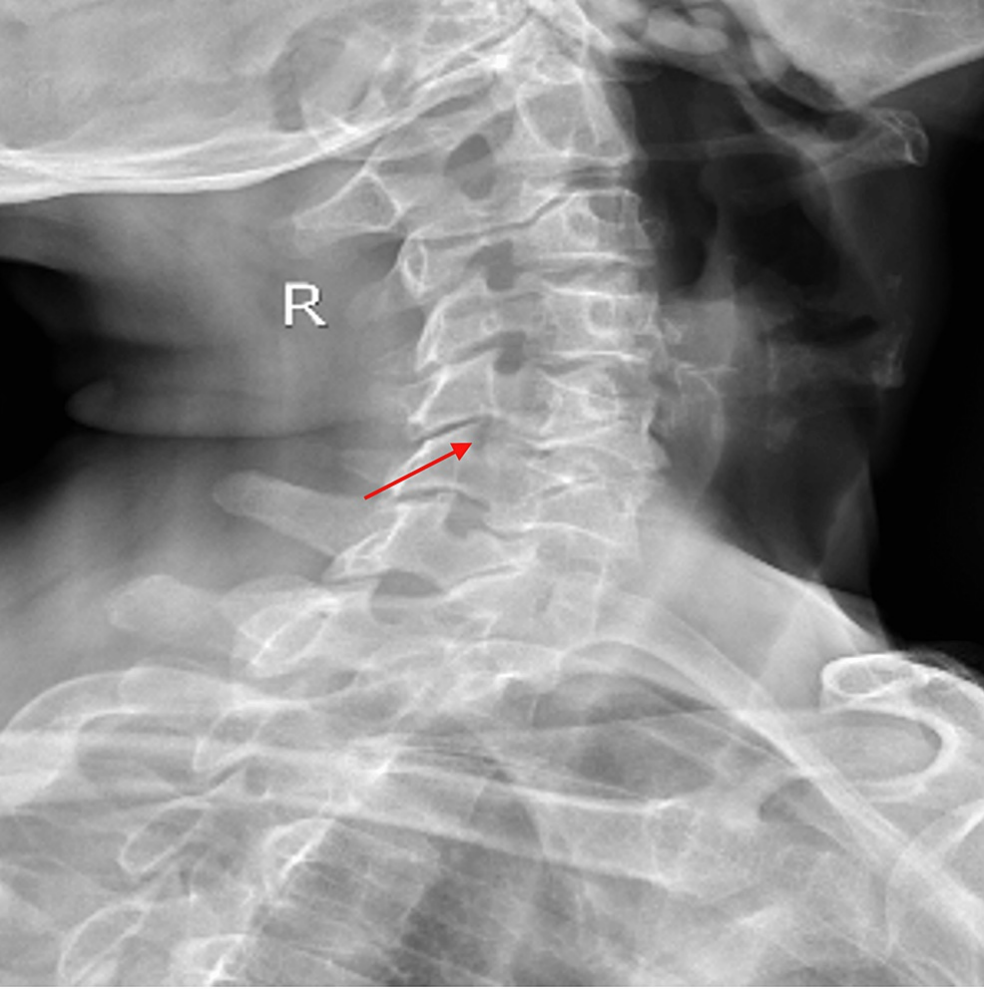
Sample X-ray image of cervical spinal stenosis

For model development, Google Collaboration, an online cloud-based AI platform, was employed. The deep learning algorithm was trained to learn various feature parameters and patterns associated with DSC-positive spine X-rays, using the labeled spine X-ray dataset (Figure 1).

Our model was trained, tested, and deployed on March 23, 2023. Our entire workflow, including the training time, was 128 minutes. 

Next, the learned feature parameters were used for training. Out of 967 total images, 773 (80%) were used for training , 97 (10%) were used for validation , and 97 (10%) were used for testing. To maintain internal validity, all assignments in this study were randomized. No external datasets were used in this study.

**Figure 2:**
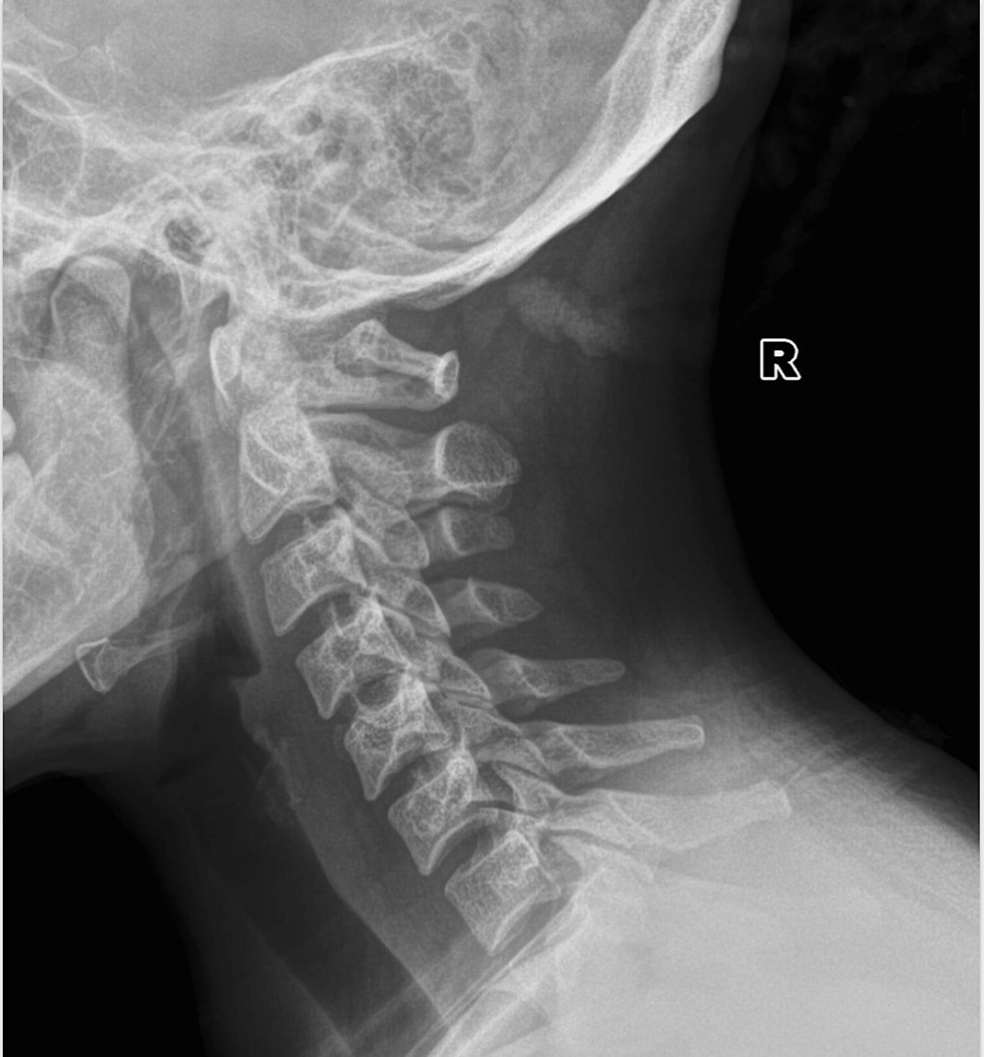
Sample X-ray image of a normal spine

To further validate the generalizability of our model performance, we conducted further testing on a separate set of spine X-ray images distinct from our aforementioned training, validation, and test data. Model performance was assessed based on its ability to accurately identify and classify DSCs into either osteophytes, spinal implant, and foraminal stenosis and distinguish them from normal X-rays (Figure 2). Diagnostic criteria were made by experienced clinicians as indicated by the dataset, but specific details were not mentioned. Evaluation metrics, including overall accuracy, confusion matrix, precision, and recall, were calculated to quantify the model performance.

The model was constructed on an Apple MacBook Air (Apple Inc., California, USA). Hardware used included an Apple M2 processing unit. Software tools used included Google Collaboration (online cloud-based AI platform), Python programming language, and various combinations of deep learning frameworks, such as TensorFlow, EfficientNet, ResNet-50, and PyTorch [14].

## Results

**Figure 3:**
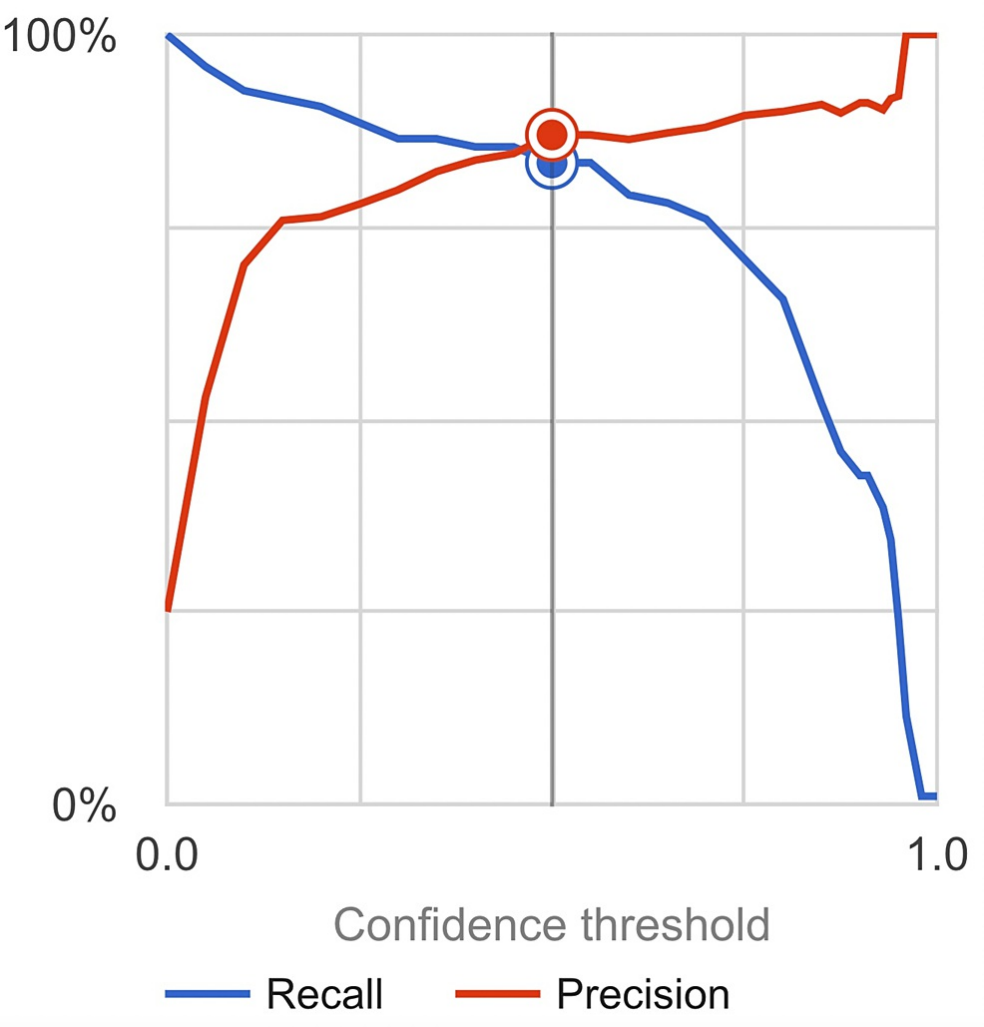
Area-under-the-curve (AUC) graph depicting the recall and precision of the model (confidence interval set to .05)

**Figure 4:**
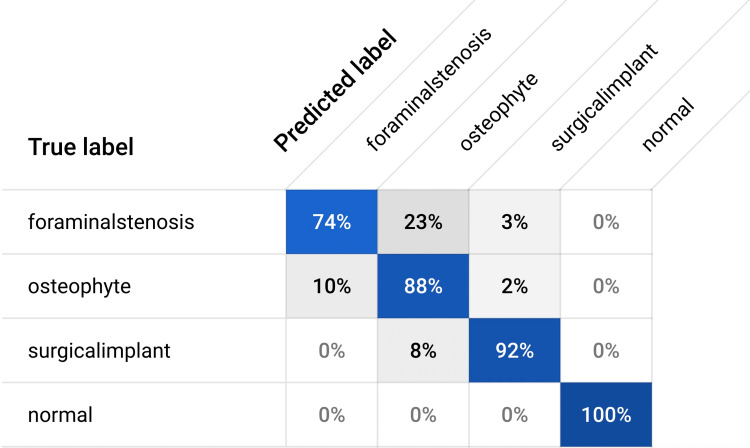
Confusion matrix demonstrating the model performance

The model developed using an online cloud-based AI demonstrated robust results. The model achieved an area-under-the-curve (AUC) value of 0.88, indicating its ability to accurately classify DSC-positive and normal spine X-ray images. Precision and recall values were found to be 87% and 83.3%, respectively (Figure 3). Sensitivity (true-positive rate) and specificity (true-negative rate) values were calculated as 94.12% and 96.68%, respectively (Table 1). These values were calculated by computing the true positive, true negative, false positive, and false negative values from the confusion matrix (Figure 4).

**Table 1:** Model performance metrics

Metric	Value
Accuracy	89%
Precision	87%
Recall	83.30%
Sensitivity	94.12%
Specificity	96.68%

The overall accuracy of the model was determined to be 89%. This value represents the percentage of all cases, both DSC-positive and normal, that were correctly identified and classified by the model. These results highlight a robust ability to accurately distinguish DSCs from normal X-ray images.

## Discussion

DSCs pose a significant global patient burden, necessitating early and accurate diagnosis for prompt management and treatment [1]. Given the current challenges of identifying and classifying DSCs, more studies are exploring the incorporation of AI and deep learning to enhance clinical workflows [9,10].

X-rays and computed tomography (CT) are widely used diagnostic modalities for DSCs [5]. In this study, our objective was to develop a model for DSC detection and classification using spinal X-ray images. Utilizing an easily accessible public dataset obtained from Kaggle.com, which included over 967 images, we trained a deep learning algorithm on an online cloud-based AI platform. The model demonstrated high precision, recall, sensitivity, specificity, and overall accuracy, indicating its effectiveness in classifying DSCs.

The model achieved an average precision of 0.88, with precision and recall values of 87% and 83.3%, respectively. Sensitivity and specificity values were calculated as 94.12% and 96.68%, respectively. The overall accuracy of the model was calculated to be 89%. These results highlight the model’s potential for aiding in the early detection of DSCs. Early diagnosis is crucial for initiating timely treatment and improving patient outcomes. Accurate DSC classification models developed through machine learning and deep learning algorithms can assist healthcare professionals in making informed decisions and potentially allocate more time for higher-level care.

Our study did have some limitations. First, the use of publicly available spinal X-ray images did not include all the variations of DSC cases. We were also unable to obtain the specific diagnostic criteria, potentially creating sources of bias. Due to the limited sample size of our dataset, we were unable to delineate our analysis into cervical, thoracic, or lumbar pathology. We also were unable to determine which spinal implants were used in our imaging sets. In addition, our initial high recall rate suggests potential overfitting, in which the algorithm is memorizing data rather than learning patterns from previous training epochs [15]. In order to correct for this, our future efforts will adjust the learning rate per epoch and increase the sample size to ensure appropriate feature learning. Although our dataset was able to achieve high accuracy with heterogeneous images from different imaging planes and spinal levels, factors, such as sample size, image capture parameters, sequence diversity, image misclassification, and choice of deep learning algorithms, may influence model performance. Our study did not evaluate the potential biases of our model toward certain patient populations or imaging modalities, which may affect accuracy and generalizability. Finally, the diagnosis of DSCs is dependent on both radiographic and clinical findings. Although our model aids in screening and diagnosis radiographically, a keen clinical workup is necessary to establish a proper diagnosis.

Future efforts will aim to recapitulate these findings in a wider variety of DSC pathology. In addition, analysis of potential model biases can help optimize our model’s performance in real-world clinical pipelines. Accounting for biases can ensure appropriate generalizability and external validity across diverse patient populations and health systems. This is best achieved with enhanced validation and deployment of our model within multiple inter-institutional cohorts of patients to critically assess clinical utility and its impact on patient outcomes.

This study demonstrates the potential of machine learning and deep learning algorithms in developing an accurate DSC screening tool for spinal X-ray images. The model exhibited high precision, recall, sensitivity, specificity, and overall accuracy, suggesting its utility in distinguishing between different types of DSCs. Continued research efforts are required to enhance the model's generalizability and facilitate its integration into clinical practice, ultimately aiding in the early detection and improved management of DSCs.

## Conclusions

This study demonstrates the potential of deep learning tools in enhancing the diagnosis of DSCs using spinal X-ray images. Our model exhibited robust accuracy and performance metrics, effectively classifying different types of DSCs and distinguishing them from normal cases. The integration of these tools into clinical workflow can potentially improve early detection, enabling prompt and enhanced management of DSCs. Multidisciplinary collaboration is paramount to improving future research, validation, and implementation efforts of similar deep learning tools. This model is a proof of concept of the ongoing technological advancements in our armamentarium for combating DSCs and minimizing their impact on global health.
